# High throughput cell-based assay for identification of glycolate oxidase inhibitors as a potential treatment for Primary Hyperoxaluria Type 1

**DOI:** 10.1038/srep34060

**Published:** 2016-09-27

**Authors:** Mengqiao Wang, Miao Xu, Yan Long, Sonia Fargue, Noel Southall, Xin Hu, John C. McKew, Christopher J. Danpure, Wei Zheng

**Affiliations:** 1 Therapeutics for Rare and Neglected Diseases, National Center for Advancing Translational Sciences, National Institutes of Health, Bethesda, MD 20892, USA; 2 West China School of Public Health, Sichuan University, Chengdu, 610041, China; 3 Department of Cell and Developmental Biology, Division of Biosciences, University College London, London WC1E 6BT, UK

## Abstract

Glycolate oxidase (GO) and alanine:glyoxylate aminotransferase (AGT) are both involved in the peroxisomal glyoxylate pathway. Deficiency in AGT function causes the accumulation of intracellular oxalate and the primary hyperoxaluria type 1 (PH1). AGT enhancers or GO inhibitors may restore the abnormal peroxisomal glyoxylate pathway in PH1 patients. With stably transformed cells which mimic the glyoxylate metabolic pathway, we developed an indirect glycolate cytotoxicity assay in a 1,536-well plate format for high throughput screening. This assay can be used to identify compounds that reduce indirect glycolate-induced cytotoxicity by either enhancing AGT activity or inhibiting GO. A pilot screen of 4,096 known compounds identified two membrane permeable GO inhibitors: dichromate salt and colistimethate. We also developed a GO enzyme assay using the hydrogen peroxide-Amplex red reporter system. The IC_50_ values of potassium dichromate, sodium dichromate, and colistimethate sodium were 0.096, 0.108, and 2.3 μM in the GO enzyme assay, respectively. Further enzyme kinetic study revealed that both types of compounds inhibit GO activity by the mixed linear inhibition. Our results demonstrate that the cell-based assay and GO enzyme assay developed in this study are useful for further screening of large compound libraries for drug development to treat PH1.

Primary hyperoxaluria type 1 (PH1, OMIM 259900) is a rare autosomal recessive disorder caused by a functional deficiency of the liver-specific peroxisomal enzyme alanine:glyoxylate aminotransferase (AGT, EC 2.6.1.44) due to mutations in the *AGXT* gene. AGT catalyzes the transamination (detoxification) of glyoxylate to glycine in liver. In PH1 patients, AGT deficiency results in accumulation of glyoxylate which is then oxidized by lactate dehydrogenase (LDH) to form oxalate^1^,^2^. The calcium salt of oxalate is highly insoluble and readily precipitates in tissues, resulting in kidney stones, kidney damage/failure, and injury to other organs. Clinically, PH1 is characterized by an increase in synthesis and excretion of oxalate and progressive deposition of insoluble calcium oxalate in the kidney and urinary tract^1^. The abnormality eventually leads to kidney failure and then calcium oxalate is deposited to almost all tissues which accounts for all the pathological characteristics of PH1 including urolithiasis, nephrocalcinosis, and systemic oxalosis. Treatment options are limited for PH1. In some patients, the disease process can be slowed by pharmacological doses of vitamin B6 (pyridoxine), a precursor of pyridoxal phosphate. It can be halted only by liver transplantation which is a highly specialized form of enzyme replacement or gene therapy^1^.

About 200 mutations in the *AGXT* gene encoding AGT have been found and the majority of them are point missense mutations^3^,^4^. These missense mutations lead to various types of protein dysfunction, including loss of catalytic activity, aggregation, accelerated proteolysis and, most remarkably, an unparalleled protein trafficking defect in which AGT is translocated to the mitochondria instead of the peroxisomes^5^,^6^. Mitochondrial AGT is metabolically inefficient because the main site of synthesis of its substrate, glyoxylate, is the peroxisome. Many of the mutations, including the most common one, segregate on the background of the minor polymorphic *AGXT* allele. The minor *AGXT* allele varies from the more common major *AGXT* allele, most significantly by the presence of a base change which encodes a Pro^11^Leu amino acid replacement. In Europeans and North Americans, < 20% of *AGXT* alleles have this polymorphic change. However, its frequency soars to over 50% in PH1 patients. Those mutations that segregate with the minor *AGXT* allele appear to require the presence of the Pro^11^Leu polymorphism in order to achieve their unfavorable effects. Gly^170^Arg is by far the most common mutation found in Caucasian patients with an allelic frequency of 30–40%. Other mutations (e.g. Ile^244^Thr and Phe^152^Ile) are also common, especially in particular patient cohorts. All three mutations together account for over half of all disease alleles and are found almost exclusively on the minor *AGXT* allele. Thus, therapeutics may be developed by stabilizing mutant AGT proteins, correcting its targeting to peroxisome, or reactivating mutant enzyme activity which can significantly clear excessive glyoxylate, the oxalate precursor.

The source of peroxisomal glyoxylate has been discussed over the years. It is likely that most is derived directly or indirectly from dietary glycolate, which is converted to glyoxylate catalyzed mainly by peroxisomal glycolate oxidase (GO, EC 1.1.3.15). Therefore, functional GO and dysfunctional AGT in PH1 patients contribute to the over productions of glyoxylate and oxalate^7^. GO is primarily expressed in liver and pancreas and is localized in peroxisomes. It catalyzes a FMN-dependent oxidation of glycolate to glyoxylate. Glyoxylate is the primary substrate for LDH to produce excessive oxalate in PH1 patients. Interestingly, glyoxylate can itself be a poor GO substrate, yielding oxalate, although this reaction may not be significantly relevant to the disease^8^,^9^. Nevertheless, the function of GO in glyoxylate production plays an important role in PH1 disease pathology. Inhibition of GO has been proposed as a potential treatment for PH1, other types of hyperoxaluria, and oxalate-mediated disorders^7^. However, previous search for GO inhibitors used the recombinant GO preparation or computational modeling that failed to identify active GO inhibitors at cellular level^7^,^10^,^11^.

Searching for small molecule AGT activators and GO inhibitors that are active at cellular level is a validated strategy for development of new therapeutics to treat PH1. We have applied a phenotypic screening approach to this task. We report here the development of a cell-based glycolate cytotoxicity assay for high throughput screening of compound libraries. A cell line stably expressing a normal GO and a mutant AGT is used to identify cell membrane permeable GO inhibitors and/or mutant AGT “activators” that include the compounds correcting the mistargeting of the mutant enzyme, stabilizing the mutant protein, or reactivating the mutant AGT. Two GO inhibitors have been identified from a screening of 4,096 FDA approved drug and known tool compounds using this cell-based assay in 1,536-well plate format. Our data demonstrate that this cell-based indirect glycolate cytotoxicity assay can be used for screening of large compound collections to identify new lead compounds for drug development to treat PH1.

## Results

### Screening assay development

**Figure 1:**
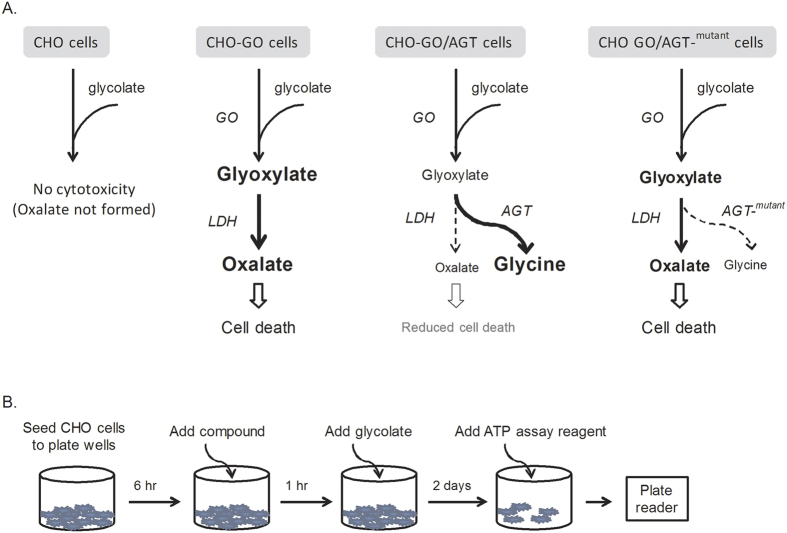
HTS using stably transformed CHO cells. (**A**) Metabolic basis of the indirect glycolate-induced cytotoxicity. (**B**) Illustration of the HTS flowchart. The HTS assay parameters were optimized (see Fig. 2).

A cell-based model with PH1 disease related metabolic events was reported previously^12^,^13^. CHO cells are particularly useful for this assay because they do not endogenously express enzymes involved in glyoxylate synthesis (e.g. GO) and detoxification (e.g. AGT). Addition of glycolate to wild type (WT) CHO cells does not confer cytotoxicity because GO is not expressed although LDH is present. This cell-based assay stably expresses GO and/or wild type (WT) or mutant AGT in CHO cells (Fig. 1A). In the transformed CHO-GO cells, glycolate is oxidized to glyoxylate in peroxisomes. Excessive glyoxylate diffuses from the peroxisomes to the cytosol to be further processed into oxalate by endogenously expressed LDH. Both glyoxylate and oxalate are toxic to cells. Addition of glycolate to the CHO-GO cells results in cytotoxicity due to the production of glyoxylate and oxalate. In CHO-GO/AGT cells, expression of WT AGT leads to conversion of toxic glyoxylate into nontoxic glycine which reduces glyoxylate level and thus leads to the decrease of oxalate level. Consequently, co-expression of WT AGT with GO significantly reduces the cytotoxicity induced by addition of glycolate. In contrast, mutated AGT in the CHO-GO/AGT-^mutant^ cells fails to reach the same protective effect as the WT AGT and thus glycolate cytotoxicity is still significantly present.

Two apparent approaches may alleviate the glycolate cytotoxicity in the CHO-GO/AGT-^mutant^ cells. One is an inhibition of GO that can prevent the production of glyoxylate from glycolate. Both CHO-GO cells and CHO-GO/AGT-^mutant^ cells can be used for this purpose to identify GO inhibitors. The other one is an enhancement of the mutant AGT activity which facilitates the clearance of glyoxylate and thus reduces its cytotoxicity. The CHO-GO/AGT-^mutant^ cells are a model system for identification of AGT mutant “activators”. A flow-chart represents the stepwise strategy to perform the screening assay in 1,536-well plate format (Fig. 1B).

### CHO-GO/AGT-152 as a model system for compound screening

**Figure 2:**
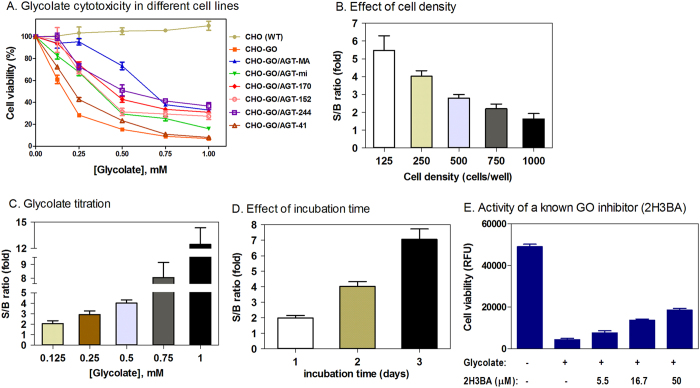
Optimization of the indirect glycolate cytotoxicity. (**A**) A collection of eight CHO cell lines were cultured in 384-well plates and assayed for the glycolate toxicity. (**B**–**D**) Optimization of assay parameters was carried out in three aspects: seeding CHO cells at different density (**B**), adding glycolate at a range of concentrations (**C**), and varying the incubation period (**D**). Signal-to-basal ratio was evaluated for each parameter. (**E**) Partial reduction of the indirect glycolate cytotoxicity in the cell-based assay by 2-hydroxy-3-butynoic acid (2H3BA) c, a known GO inhibitor.

In order to choose the appropriate AGT mutants for compound screening assay, we first evaluated several alleles of AGT mutations (details in the Discussion). We examined six CHO cell lines expressing WT GO and normal or mutated AGT (AGT-MA and AGT-mi; AGT-170, AGT-152, AGT-244, and AGT-41 all in the background of AGT-mi) (Fig. 2A) in a glycolate cytotoxicity assay. Addition of glycolate resulted in cell death in CHO-GO cells but not in untransformed CHO WT cells (Fig. 2A). Co-expression of AGT-MA significantly reduced the cytotoxicity of glycolate, while co-expression of AGT-mi, AGT-170, AGT-152, or AGT-244 partially reduced the cytotoxicity (Fig. 2A). Among these partially functional AGT mutants, AGT-152 gives the largest assay window at a final concentration of 0.25 or 0.5 mM glycolate. In contrast, co-expression of AGT-41 provided little cellular protection, suggesting that this AGT mutant is largely nonfunctional. Therefore, we selected the CHO-GO cell line and CHO-GO/AGT-152 cell line as a model system for respective identification of GO inhibitors and AGT mutant activators.

### 1,536-well indirect glycolate cytotoxicity optimization using CHO-GO/AGT-152 cells

To optimize the indirect glycolate cytotoxicity assay in 1,536-well plate format, we first determined the effect of cell density on the assay signal-to-basal (S/B) ratio (Fig. 2B). The S/B ratios were 5.47 ± 0.82, 4.03 ± 0.30, 2.80 ± 0.20, 2.20 ± 0.26, and 1.63 ± 0.30 respectively for 125, 250, 500, 750, and 1,000 cells plated per well. As higher cell densities (>500 cells/well) resulted in smaller assay windows, we decided to use 250 CHO cells per well in 1,536-well plates for further experiments. We then compared the effect of glycolate concentration on the assay window (Fig. 2C). The S/B ratios were 2.07 ± 0.27, 2.92 ± 0.35, 4.03 ± 0.30, 8.04 ± 1.10, and 12.45 ± 1.90 respectively for final glycolate concentrations of 0.125, 0.25, 0.5, 0.75, and 1 mM. We selected 0.5 mM glycolate for further experiments since this final concentration resulted in sufficient S/B with appropriate cytotoxicity. Finally, we tested different lengths of incubation time after glycolate addition (Fig. 2D). The S/B ratios were 1.99 ± 0.16, 4.03 ± 0.30, and 7.06 ± 0.68 respectively for 1, 2, and 3 day incubation. Two days incubation with glycolate was selected for this compound screening assay because it yielded an appropriate S/B ratio. Taken together, we optimized the compound screening conditions for the indirect glycolate cytotoxicity using the CHO-GO/AGT-152 cell line with 250 cells/well and 2-day incubation with 0.5 mM glycolate. Using this optimized condition, we tested 2-hydroxy-3-butynoic acid (2H3BA), a known GO inhibitor^14^,^15^. This compound partially and weakly reduced the indirect glycolate cytotoxicity (Fig. 2E). The result demonstrates that this cell based assay has a capability to identify GO inhibitors from compound screening.

### Compound screening with indirect glycolate cytotoxicity using CHO-GO/AGT-152 cells

**Figure 3:**
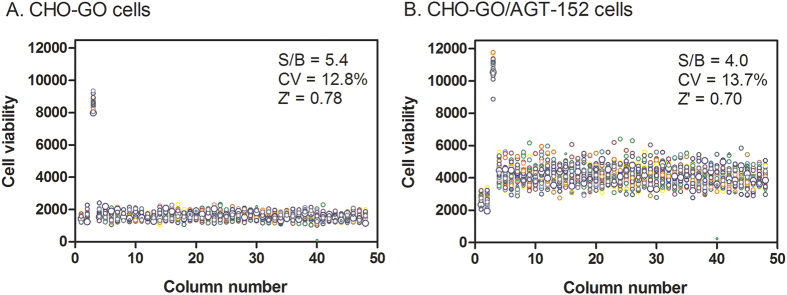
Scatter plots of DMSO screening plates. The signal-to-basal ratio (S/B), coefficient of variation (CV), and Z’ factor were calculated and displayed. The wells in column 3 contained untransformed CHO WT cells while the wells in column 1 and 2 contained CHO-GO cells. GO DMSO assay plate contained CHO-GO cells in columns 4–48 (**A**), and GO/AGT-152 DMSO assay plate contained CHO-GO/AGT-152 cells in columns 4–48 (**B**). All wells were added with 23 nl DMSO for 1 h followed by glycolate addition (final glycolate concentration of 0.5 mM and DMSO concentration of 0.57%).

First we tested the assay performance under a compound screening condition. After cell seeding and culture in 1,536-well plates, compounds were added 1 h before glycolate addition to allow compound diffusion into cells. The compound screening protocol included four cell/reagent addition steps and four incubation periods without any plate-washing steps (Table 1). Two types of cells were used in HTS including CHO-GO cells for identification and confirmation of GO inhibitors and CHO-GO/AGT-152 cells for discovering AGT-152 activators. A DMSO plate (without compounds) was tested to assess the parameters of assay performance. For CHO-GO cells, the S/B ratio was 5.4-fold, the coefficient of variation (CV) was 12.8%, and Z´ factor was 0.78 determined in the DMSO plate (Fig. 3A). The S/B ratio was 4.0-fold, the CV was 13.7%, and Z´ factor was 0.70 as obtained in the DMSO plate of CHO-GO/AGT-152 cells (Fig. 3B). Thus, both cell lines produced robust assay windows and Z’ factors which are suitable for HTS.

We then performed the primary screening using compounds from two compound libraries: (1) the Library of Pharmacologically Active Compounds (LOPAC) and (2) the NCATS Pharmaceutical Collection (NPC). All compounds in both libraries were serially diluted for 4 concentrations that resulted in a final compound concentration of 0.46, 2.3, 11.5, and 57.5 μM in the assay plates. Cell death occurred after the CHO-GO/AGT-152 cells were treated with 0.5 mM glycolate due to the cytotoxicity of generated glyoxylate and oxalate (Fig. 1A). The cell death would decrease if a GO inhibitor was present and this screening was to identify compounds that reduced cell death caused by 5 mM glycolate added to the CHO-GO/AGT-152 cells. Since the dose-response was employed in the primary compound screening, we were able to pick up the hits based on their potency, the compound-centration producing 50% of maximal response (IC_50_) and maximal response (maximal increase in cell viability). The primary hits were then selected using the cut-off criteria of IC_50_ <40 μM and maximal inhibition >20%. A total of 10 compounds (7 from the LOPAC and 3 from the NPC) were selected as primary hits and subjected to confirmation tests.

### Counter-screen and confirmation of primary hits

The selected 10 primary hits were re-screened to confirm compound activity in the same cell lines used in the primary screen. In this secondary screen, six compounds were found to reduce glycolate cytotoxicity in both CHO-GO and CHO-GO/AGT-152 cells (Table 2). None of the six hits were able to preferentially reverse the glycolate cytotoxicity in CHO-GO/AGT-152 cells. The IC_50_ values of these compounds in CHO-GO/AGT-152 cells were similar to those in CHO-GO cells (within 2-fold), suggesting that the mechanism of action for these compounds is mediated by a direct inhibition of GO but not by activating mutant AGT (Table 2). Taken together, the quantitative HTS yielded six compounds that may block glycolate toxicity in CHO-GO cells by a direct inhibition of GO. AGT mutant activators were not found in this screen.

### Confirmation of three active compounds in GO enzymatic assay

**Figure 4:**
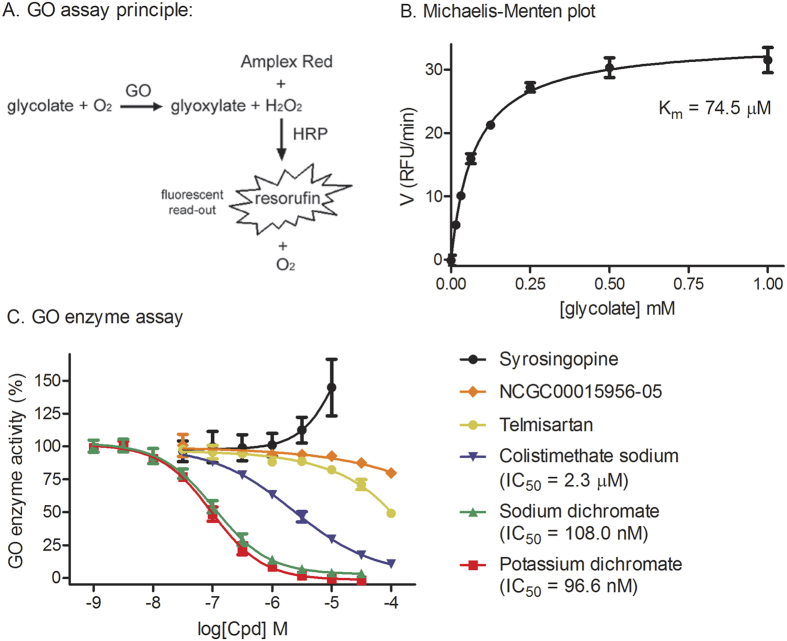
Confirmation of HTS hit compounds. (**A**) Mechanism of the *in vitro* GO enzymatic assay. Recombinant GO oxidizes glycolate to glyoxylate, and hydrogen peroxide is a co-product. Amplex red reagent reacts with hydrogen peroxide in a 1:1 stoichiometry and produces fluorescent resorufin. The resulting fluorescent signal is an accurate linear reflection of the initially available H_2_O_2_, which is itself a linear reflection of the *in vitro* GO enzymatic activity. (**B**) The Michaelis-Menten plot for the *in vitro* GO enzymatic assay. (**C**) Six HTS hit compounds were tested in the GO enzymatic assay. Compounds were added at a series of doses to purified GO for 10 min followed by the addition of glycolate to start the reaction. After another 10 min, Amplex red reagent was added and the wells were evaluated for fluorescence signal. Signal of GO without addition of compound was defined as 100% response while signal of GO without addition of glycolate was defined as 0% response.

To investigate if the six hit compounds directly inhibit GO, we developed a GO enzymatic assay using the hydrogen peroxide-Amplex red reporter system that is described in Fig. 4A. The K_m_ value of glycolate for GO in this enzyme assay was 74.5 μM (Fig. 4B). To determine the inhibitory mechanism of action for these compounds, a series of compound concentration was added into the GO enzyme kinetic assay (Fig. 4C). The GO enzymatic assay confirmed three compounds that directly and efficiently inhibit GO enzymatic activity including potassium dichromate (IC_50_ = 96.6 nM), sodium dichromate dihydrate (IC_50_ = 108.0 nM), and colistimethate sodium (IC_50_ = 2.3 μM). All three compounds produced sigmoidal dose-response curves. Similar to their weak effect as observed in CHO-cell-based assay, the other two compounds of SB 222200 and telmisartan only partially inhibit GO enzymatic activity (less than 50% inhibition at 100 μM). The 6th compound syrosingopine did not inhibit GO enzymatic activity in this assay. We then observed this compound has self-fluorescence that could be the reason as a false positive compound. Together, three compounds (potassium dichromate, sodium dichromate dihydrate, and colistimethate sodium) identified from the primary screen were confirmed as GO inhibitors in this GO enzymatic assay.

### Inhibitory mechanism of three GO inhibitors

**Figure 5:**
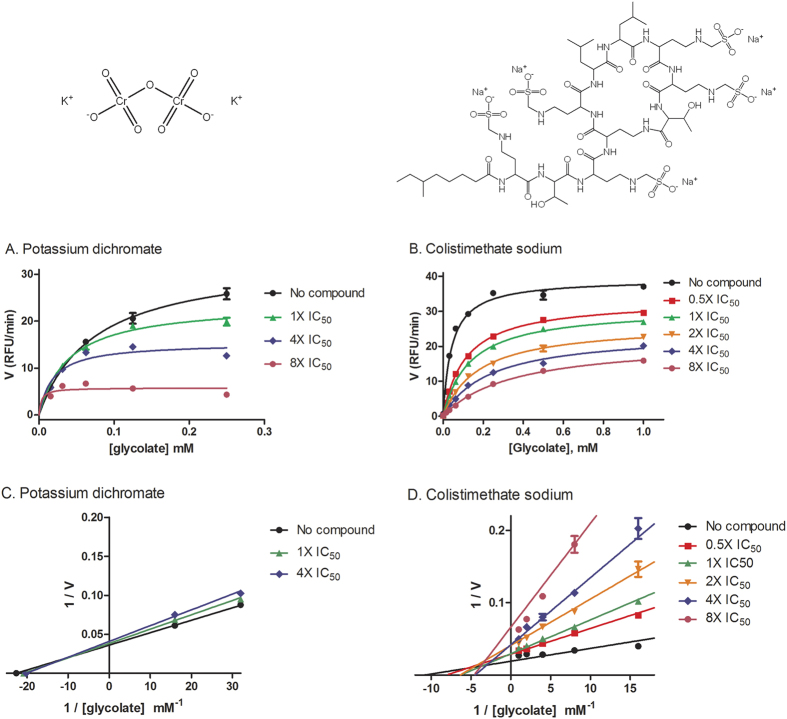
Characterization of the mechanism of novel GO inhibitors. (**A**,**B**) For the confirmed GO inhibitors, various doses (1X, 4X, and 8X of IC_50_) of potassium dichromate (**A**) or sodium dichromate dihydrate, or various doses (0.5X, 1X, 2X, 4X, and 8X of IC_50_) of colistimethate sodium (**B**) were added to purified GO, and the time-series signal of the *in vitro* GO enzymatic assay was monitored to obtain the reaction velocity for each glycolate concentration tested. For conciseness, data for sodium dichromate dihydrate is not shown since it can be represented by data for potassium dichromate. (**C**,**D**) The Lineweaver-Burk curves (also called the double reciprocal curves) were accordingly plotted for potassium dichromate (**C**) and colistimethate sodium (**D**).

To investigate the inhibitory mechanism of confirmed GO inhibitors, we measured the GO enzyme kinetics in the presence of various inhibitor concentrations. These three compounds are actually two types in chemical structures. Potassium dichromate and sodium dichromate dihydrate share the common structure of dichromate and are different salts. Both compounds inhibited GO activity at higher substrate concentrations (Fig. 5A). In comparison, colistimethate sodium inhibited GO activity at all concentrations of substrate (Fig. 5B). The Lineweaver-Burk curve was plotted and the position of the crossing point can be used to suggest the potential inhibitory mechanism for the compounds. Competitive inhibition would have the crossing point on the Y (1/V) axis and non-competitive inhibition would have the crossing point on the X (1/[S]) axis. The crossing points were neither on the Y-axis nor X-axis for all three compounds (Fig. 5C,D), suggesting that the mechanism of action of these compounds is the mixed linear inhibition.

**Figure 6:**
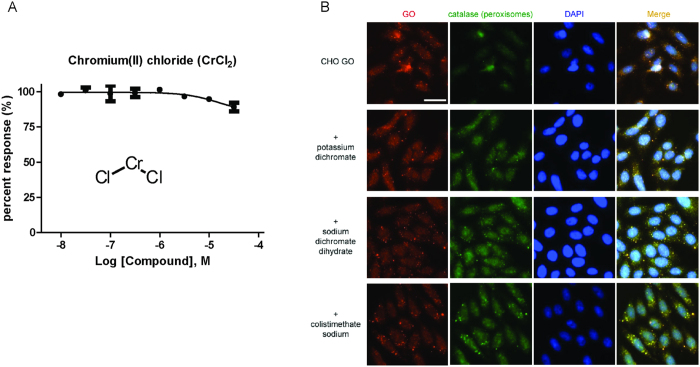
(**A**) Concentration-response of chromium(II) chloride (450782, Sigma-Aldrich) determined in the GO enzymatic assay. (**B**) Immunofluorescence images of GO localized in peroxisomes. CHO GO cells were treated with 100 μM potassium dichromate, sodium dichromate dihydrate, or colistimethate sodium for 2 h, then immunostained for GO, the peroxisomes (catalase) and the nucleus (DAPI), and finally analyzed by fluorescence microscope (Scale bar, 30 μm). Compound treatment did not significantly change the localization and amount of GO in peroxisome.

Both potassium dichromate and sodium dichromate dihydrate share a functional group in structure. In comparison to the dichromate (Cr_2_O_7_^2−^) group, chromium(II) chloride, which has Cr^2+^, does not inhibit GO enzymatic activity (Fig. 6A). The result suggests unique structural and chemical features of the dichromate group in this function. In addition, GO is specifically targeted to the peroxisomes. Consequently, authentic GO inhibitors should inhibit GO functional activity but should not change its cellular localization. We found that GO were physiologically present in the peroxisomes of CHO-GO cells without significant change after treatment with the 100 μM compound which produced the maximal inhibition of the glycolate toxicity (Fig. 6B), further demonstrating the direct functional inhibition of GO by these three compounds.

## Discussion

Since PH1 is caused by the genetic mutations of AGT enzyme, identifications of AGT mutant enzyme activators and GO inhibitors are two potentially therapeutic approaches for small molecule drug development to treat this rare disease. Cell-based phenotypic screening is a new approach^16^ to identify AGT activators or GO inhibitors that can penetrate into cell membrane and are active in cells. We have optimized a cell-based assay using cell lines expressing GO and mutant AGT for high throughput screening of compound collections to identify lead compounds. In our phenotypic screening of known compound libraries, we identified two types of GO inhibitors that significantly ameliorate indirect glycolate-induced cytotoxicity in the cells expressing GO and mutant AGT. The mutant AGT activators, if they are identified by this cell-based assay, might be useful only in PH1, while GO inhibitors would be useful in PH1 and PH2 as well as some potential value in PH3.

The majority of normal population has Pro^11^ for AGT and this allele is considered the major allele (AGT-MA). A Pro^11^Leu polymorphism is present with an allelic frequency of 5–15% in the normal Caucasian population and represents a key feature of the minor allele (AGT-mi). In comparison to the major allele, AGT-mi has an N-terminal amphiphilic α-helix leading to a weak mitochondrial targeting sequence^17^. Therefore, individuals homozygous for the minor allele target about 5% of their AGT to the mitochondria where AGT is presumed to be inefficient to metabolically detoxify glyoxylate^18^. Four most common AGT point mutations segregate with Pro^11^Leu and work synergistically with the minor allele to render AGT nonfunctional^13^. AGT-170 (Gly^170^Arg) and AGT-152 (Phe^152^Ile) are both defective in peroxisomal targeting and/or import^17^. AGT-244 (Ile^244^Phe) undergoes conformational change and protein misfolding^19^. AGT-41 (Gly^41^Arg) appears to aggregate as core-like structures within the peroxisome matrix with little or no catalytic activity^17^. These mutations cause the clinically significant manifestations of the PH1 disease.

The reconstituted glyoxylate metabolic pathway in stably transformed CHO cells provides a target-based screening platform. Approaches to reduce the biosynthetic output of oxalate could result from either limiting the production of glyoxylate from its precursor of glycolate or preventing the conversion of glyoxylate to oxalate. In our cell-based assay, potentially potent AGT activators would be able to restore the function of mutant AGT in cells expressing mutant protein which results in a reduction of cytotoxicity induced by glycolate. Deficiency in AGT function due to various mutations can arise from the incorrect folding or processing, instability, aggregation, and mistargeting of this mutant protein to mitochondria instead of peroxisomes^20^. Thus, a compound that can correct these pathological changes of mutant protein may enhance or restore AGT function. However, targeted activation of enzymes through allosteric binding site may not be easy to achieve which is especially more difficult in the cellular context, thus making it a tendency that physical binding of small molecules would inhibit rather than enhance enzyme activity^21^. Generally, an enzyme activator is much more difficult to be discovered than an enzyme inhibitor. We encountered the exact same situation in our screening of ~4,000 known compound collections as we found 10 primary hits as GO inhibitors without any AGT functional activators. Pyridoxine^22^ and dequalinium chloride^23^ were previously reported to partially restore mutant AGT function by correction of the trafficking defect of mutant AGT. However, both compounds appeared inactive in our screening although they were present in the compound collections we screened. The cell culture medium in our assay contains 291 μM pyridoxine and thus this assay is insensitive to this compound. Dequalinium chloride partially rescued the trafficking defect of AGT mutant enzymes^23^. Our cell-based assay measures the activity of compounds that reduce glycolate-induced cytotoxicity in the engineered CHO cells that is different from these enzyme translocation assays^22^,^23^ reported previously. Weak AGT activators or translocators/chaperone compounds may not be identified in our assay because they may not be able to block the cell death due to glycolate-induced cytotoxicity in the engineered CHO cells. After addition of glycolate, oxalate level in CHO AGT-170 was approximately 2.7-fold of that in CHO AGT-WT^23^. It reduced to 2-fold after the treatment with dequalinium chloride^23^. While a reduction of oxalate from 2.7-fold to 2-fold could be significant in the assay previously reported, dequalinium chloride could not rescue the glycolate-induced cytotoxicity in our assay because oxalate level remained high. It may suggest that our cell-based assay may not be sensitive enough to identify weak activators of AGT mutants or a future screening of much larger compound collections is needed to increase the chance for identification of enzyme activators. In addition, the AGT-152 used here is only one of many mutations found in this disease. The screening results may depend on the respective AGXT mutations used. Thus, more mutant cell lines should be generated for compound screening to examine whether additional hits can be identified.

Our screen yielded six primary hits that suppress the glycolate toxicity with similar IC_50_ values in both CHO-GO and CHO-GO/AGT-152 cells. In order to examine their direct activity on the GO enzyme, an appropriate enzyme assay is needed. However, it is challenging to accurately quantify the amount of enzyme reaction product glyoxylate in a GO enzyme assay. Since GO converts substrate glycolate to glyoxylate with the co-product of hydrogen peroxide, we have developed a new GO enzyme assay using the hydrogen peroxide-Amplex red reporter system that detects H_2_O_2_ formation. Similar assay design has been utilized to measure the activity of other oxidases including glucose oxidase, cholesterol oxidase, and monoamine oxidase^24^,^25^,^26^. In our GO enzyme assay, the Km of glycolate for human GO (74.5 μM) is comparable to the Km of glycolate for GO purified from human (56 μM)^9^, pumpkin seedlings (330 μM)^27^ or rice leaves (400 μM)^28^. This fluorogenic GO enzyme assay is homogeneous with high sensitivity, allowing a detailed study of enzyme kinetics and compound inhibition mechanism. This GO enzyme assay is also robust and can be miniaturized to 1,536-well plates for high throughput screening of large compound collections.

Among 6 primary hits examined in the GO enzyme assay, we confirmed three compounds with the GO inhibitory activity. These three novel GO inhibitors can be categorized into two different types. Potassium dichromate and sodium dichromate dihydrate are different forms of dichromate which are commonly used as oxidizing agents in laboratory and industrial applications. Colistimethate sodium, a form of the polymyxin antibiotic of colistin, disrupts the bacterial cell membrane and is used for treating multidrug-resistant bacteria infections^29^,^30^. The inhibition of GO by dichromate compounds is more significant at higher concentration of substrate (over 30 μM of glycolate), while colistimethate sodium appears to inhibit GO enzymatic activity throughout the concentrations of glycolate tested. Although both inhibitors have similar maximal activity that completely inhibit GO activity, the potency of dichromate compounds (IC_50_ = ~0.1 μM) is 20 times higher than that of colistimethate sodium (IC_50_ = 2.3 μM). A few GO inhibitors were reported previously including dichlorophenol indophenol (DCIP) (IC_50_ = 33–50 μM)^9^, TKP and TACA^11^, and CCPST^31^. These compounds are generally weak enzyme inhibitors and not potent or active in the cell-based assays.

Because library compounds are almost exclusively prepared in DMSO, compound solubility in DMSO could significantly affect its activity^32^. During our confirmation study of the primary hits, we found the two dichromate compounds precipitated in DMSO at the concentration over 3 mM. Their apparent IC_50_ values in the CHO-cell-based assay were in micromolars using the DMSO solution. However, when both compounds were prepared in water, dichromate is soluble up to 100 mM. Their IC_50_ values were correspondingly lowered by approximately 100 fold in the same assay compared to these obtained using the DMSO compound solutions.

GO inhibitors have a therapeutic potential for the treatment of PH1 and other forms of hyperoxaluria. For example, 2-hydroxy-3-butynoic acid, a GO inhibitor, was patented for the treatments of urinary tract diseases, especially for renal calcium oxalate lithiasis (https://www.google.com/patents/US4178386 and http://www.google.com/patents/US4428956). GO inhibitors would significantly reduce the intracellular level of glyoxylate and oxalate, and facilitate the re-balance of the glyoxylate pathway in PH1 patients. Notably, the conversion of glycolate to glyoxylate by GO accompanies the production of hydrogen peroxide in the peroxisomes. Hydrogen peroxide is a strong oxidant which must be immediately broken down to water and oxygen by the enzyme catalase. Cells with the catalase mutations suffer accumulation of hydrogen peroxide that ultimately leads to oxidative stress and damage^33^. GO inhibitors could reduce the production of hydrogen peroxide and thus decrease the oxidative damage. In addition, GO is also present in the peroxisomes of plants and is a key player of oxidation of glycolate to glyoxylate in photorespiration^34^,^35^. In plants, glycolate oxidase (GOX) is involved in photorespiration via the formation of phosphoglycolate in the oxygenase reaction of ribulose-1,5-bisphosphate carboxylase/oxygenase (Rubisco)^36^. Net photosynthesis is drastically reduced due to this pathway for carbon metabolism. Thus, GOX inhibition can significantly increase photosynthetic CO_2_ uptake^37^.

In conclusion, we have developed and optimized the cell-based screening assay for compound screening to identify AGT activators and GO inhibitors. Due to the limitations of the mutations used and the nature of cell-based model, some previously reported GO enzyme inhibitors (such as dequalinium chloride) were not identified in this cell-based GO assay. This assay has been validated in a pilot screening of approximately 4,000 known compounds that identified two novel GO inhibitors. Therefore, this assay can be used for the further screening of large compound collections to identify new lead compounds. This cell-based assay and phenotypic screening approach described here provide a new direction for drug development to treat PH1 disease.

## Methods

### Cell lines

Details of the expression constructs and the Chinese hamster ovary (CHO) cell lines were as reported previously^13^. Briefly, full-length cDNAs of GO and AGT were respectively sub-cloned into the mammalian expression vectors of pcDNA3.1(+)neo (Invitrogen) and pcDNA3.1(−)zeo (Invitrogen). CHO cell lines expressing AGT variants were established by retransforming a CHO cell line previously transformed with GO to form CHO-GO/AGT cells. Two cell lines expressed normal AGT variants: AGT-MA expressing AGT encoded by the major allele of *AGXT*, and AGT-mi expressing AGT encoded by the minor allele of *AGXT*. Four cell lines expressed mutated alleles of AGTs: AGT-170, AGT-152, AGT-244 and AGT-41. Untransformed CHO wild type (WT) cells were included as control. Initial characterization involved 8 CHO cells lines (WT, GO, 2 GO/AGT normal variants, and 4 GO/AGT mutants). After comparison, the CHO-GO (GO only control) and CHO-GO/AGT-152 cell lines were used for the HTS as they produced better assay signals.

### Cell culture

Untransformed CHO cells were cultured in Ham’s F-12K media (21127-022, Gibco) supplemented with 10% fetal bovine serum (FBS), and 50 units/ml penicillin/50 μg/ml streptomycin (15070-063, Gibco). CHO cells stably transformed with vector expressing GO (CHO-GO) were cultured with additional supplement of 400 μg/ml Zeocin (R25001, Life Technologies). CHO cells stably transformed with vectors expressing both GO and AGT (CHO-GO/AGT) were cultured with additional supplement of 400 μg/ml Zeocin and 800 μg/ml G418/Geneticin (10131035, Life Technologies). Cells were cultured and passaged in T-75 flasks, and placed in a humidified incubator with 5% CO_2_ at 37 °C.

### Compound libraries

The library of pharmacologically active compounds (LOPAC) is a collection of 1,280 small molecules associated with known biological activities and purchased from Sigma-Aldrich. The NIH Chemical Genomics Center pharmaceutical collection (NPC) consists of 2,816 small molecule compounds, 52% of which were drugs approved for human or veterinary use by the United States Food and Drug Administration (FDA), 22% were drugs approved in Europe, Canada or Japan, and the remaining 25% were compounds that entered clinical trials or were research compounds commonly used in biomedical research. The NPC library was constructed internally at NIH^38^. Compounds from both libraries were dissolved in DMSO as 10 mM stock solutions, except for several hundred compounds from the NPC library that were dissolved as 4.47 mM stock solutions due to solubility issues. All library compounds were serially diluted in DMSO by 1/5 inter-plate titrations for primary screen, and then formatted to 1,536-well compound plates (Greiner Bio-one) using an Evolution P3 system (PerkinElmer). Compound plates were sealed and stored at room temperature in desiccated containers. For the hit confirmation, compounds were prepared as 10 mM solutions (except for a few NPC library compounds that are prepared as 4.47 mM solutions), diluted in DMSO as 11-points of 1/3 dilutions from the highest compound concentration, and then formatted to 1,536-well compound plates (Greiner Bio-one). We observed solubility issue of potassium dichromate and sodium dichromate dihydrate in DMSO. Therefore, we repeated the assay in CHO-GO cells using these two compounds dissolved in distilled water.

CHO-GO and CHO-GO/AGT-152 cells were added with serially diluted concentrations of hit compounds and analyzed for their glycolate toxicity. Syrosingopine was diluted at 11 concentrations (1/3 dilution from the highest compound concentration, 0.4 nM–26 μM); the other five compounds were diluted at 11 concentrations (1/3 dilution, 1 nM–57 μM). Signal of untransformed CHO WT cells added with DMSO was defined as 100% response. Signal of CHO-GO or CHO-GO/AGT-152 cells added with DMSO were defined as their respective 0% response.

### Cell-based indirect glycolate cytotoxicity in 384-well plate format

To compare the glycolate toxicity in all 8 CHO cell lines, cells were plated in white solid-bottom 384-well plates (Greiner Bio-one) at an empirical seeding density of 1,000 cells in 20 μl media per well using the Multidrop Combi dispenser (Thermo). After incubation at 37 °C with 5% CO_2_ for 6 h, 5 μl of 4 mM glycolate (pH 7.4), prepared freshly in culture media from 30 mM stock solution (pH 7.4), was added to the assay plates. The plates were then incubated at 37 °C with 5% CO_2_ for a period of 2 days before being assayed by an ATP content cell viability assay using an assay kit (21610, AAT Bioquest). Briefly, 25 μl ATP detection reagents prepared as described in the manufacturer’s protocol were added to each well in the assay plates. The reaction was incubated at 37 °C for 10 min, and then the luminescence signal was read in a luminescence detection mode on a ViewLux plate reader (PerkinElmer).

### Cell-based indirect glycolate cytotoxicity assay in 1,536-well plate format

Quantitative HTS of the cell-based indirect glycolate cytotoxicity was conducted in white solid-bottom 1,536-well plates (Greiner Bio-one). Assay optimization was carried out by determination of the optimal conditions including cell seeding density, substrate (glycolate) concentration, and incubation time. CHO cells were plated at a seeding density of 125, 250, 500, 750 and 1,000 cells in 3 μl media per well using the Multidrop Combi dispenser (Thermo). After incubation at 37 °C with 5% CO_2_ for 6 h, 1 μl glycolate, prepared freshly in culture media from 20 mM stock solution (pH 7.4) to a concentration range from 0.5 to 4 mM, was added to the assay plates. The plates were then incubated at 37 °C with 5% CO_2_ for a period of 1, 2 or 3 days before being assayed by the ATP content cell viability assay as described above. Briefly, 3 μl ATP detection reagents were added to each well in the assay plates. The reaction was allowed to proceed at 37 °C for 10 min, and then the luminescence signal was read in a ViewLux plate reader. After evaluation of data from the optimization experiments, optimal parameters were determined for cell seeding density, glycolate concentration, and incubation time. All following experiments including the HTS are performed at the optimal assay conditions. For HTS, the indirect glycolate cytotoxicity is similar as above, except that compounds need to be added before glycolate addition. Briefly, after dispensing 3 μl cells into 1,536-well plates and incubation at 37 °C with 5% CO_2_ for 6 h, 23 nl compounds from the library plates were added to the assay plates by an automated pin-tool workstation (Wako-Kalypsys). After additional 1 h incubation at 37 °C, 1 μl glycolate of optimal concentration was added to the assay plates, and the remaining steps are similar as above.

### *In Vitro* GO enzymatic assay

The recombinant full-length human hydroxyacid oxidase 1 (HAO1), the equivalent of GO, was purified using conventional chromatography to >95% purity (ab113144, AbCam). Purified HAO1 was used for *in vitro* GO enzymatic assay and dissolved in assay buffer (10 mM NaCl, 110 mM KCl, 2 mM MgCl_2_, 50 mM HEPES pH 7.4, 10 μM DL-dithiothreitol, 0.01% Triton X-100). 20 μl 50 nM HAO1 and 20 μl 360 μM glycolate (pH 7.4) were added into wells of black, solid-bottom 384-well plates (Greiner Bio-one). Thus, the final concentration is 25 nM HAO1 and 180 μM glycolate. Gently rotate the plate to allow mixing and incubate at room temperature for 10 min. Then add 40 μl Amplex red reagent (A12222, Invitrogen) of 2 unit horseradish peroxidase (HRP) and 300 μM Amplex red dye to each well, and incubate at room temperature for 10 min. Fluorescence signal was read by ViewLux plate reader (PerkinElmer) with Ex = 560 ± 10 nm and Em = 590 ± 10 nm. For inhibition of HAO1 with identified hit compounds, preferred concentrations were added in a 1:100 dilution to HAO1 and incubated for 10 min before addition of glycolate. Given the fast speed of reaction between HAO1 and glycolate, for time-series enzymatic assay, Amplex red reagent was added first and followed immediately by the addition of glycolate; the reaction was repeatedly measured for fluorescence signal every 1 min, and data from the first 8 min were used to plot and calculate the reaction velocity values.

### Immunostaining and fluorescence microscopy

CHO cells were cultured and plated at 3,000 cells in 100 μl media per well into black, clear-bottom 96-well plates (Greiner Bio-one). After overnight incubation at 37 °C and 5% CO_2_, cells were treated accordingly with compounds, and subjected to immunostaining for catalase and GO. Briefly, cells were fixed in 4% paraformaldehyde for 20 min, rinsed with PBS, permeabilized with 0.3% Triton X-100 for 15 min, and followed by blocking with cell staining buffer (420201, BioLegend) for 60 min. Cells were then incubated with 1:50 dilution of primary antibodies against catalase (sc-50508, Santa Cruz Biotechnology) and against GO (sc-85589, Santa Cruz Biotechnology) at room temperature for 2 h. After washing with PBS, anti-rabbit secondary antibody conjugated with Alexa Fluor 488 (A11034, Life Technologies) and anti-goat secondary antibody conjugated with Alexa Fluor 594 (A11058, Life Technologies) were added in 1:250 dilution for 1 h. Cells were then stained for the nucleus with Hoechst 33342 (H3570, Life Technologies) for 20 min and finally imaged for tri-color fluorescence using INCell Analyzer 2000 (GE Healthcare) with 40x objective lens, and through FITC, Texas Red and DAPI filter sets. Microscopy images were processed in INCell Analyzer software (GE Healthcare).

### Data analysis

The primary screen data was analyzed using customized software developed internally^39^. IC_50_ values (the half maximal inhibitory concentration) from confirmation experiments were calculated from dose-response signal curves using the Prism software (GraphPad Software). Signal-to-basal (S/B) ratio was calculated as the signal of untransformed CHO WT cells divided by the signal of the stably transformed CHO cells (CHO-GO or CHO-GO/AGT-152). Coefficient of variation (CV) was defined as the ratio of the standard deviation to the mean. The Z’ factor index of assay quality control measured the statistical effect size, and was calculated by the formula of Z’ = 1–3(SD_Total_ + SD_Basal_)/(Mean_Total_ − Mean_Basal_)^40^, where SD_Total_ and Mean_Total_ are the standard deviation and mean of signal for untransformed CHO WT cells, and SD_Basal_ and Mean_Basal_ are the standard deviation and mean of signal for stably transformed CHO cells (CHO-GO or CHO-GO/AGT-152). Data normalization and curve fitting was performed as previously reported^41^. Briefly, signal of untransformed CHO WT cells added with DMSO was defined as 100% response. Signals of CHO-GO or CHO-GO/AGT-152 cells added with DMSO were defined as their respective 0% response. Assays were performed in triplicate, and standard errors of the mean were shown. As an exception for the GO enzymatic assay, variation was very small and instead standard deviations were shown.

## Additional Information

**How to cite this article**: Wang, M. *et al*. High throughput cell-based assay for identification of glycolate oxidase inhibitors as a potential treatment for Primary Hyperoxaluria Type 1. *Sci. Rep*. **6**, 34060; doi: 10.1038/srep34060 (2016).

## Figures and Tables

**Table 1:** Protocol of the indirect glycolate cytotoxicity adapted for quantitative HTS in 1,536-well plate format.

Step	Description	Parameter value	Note
1	Cell plating	250 cells in 3 μl/well	re-suspend cells to 8.3 × 104 cells/ml
2	Incubation	6 h	at 37 °C and 5% CO2
3	Compound addition	0.023 μl/well	compound in DMSO
4	Incubation	1 h	at 37 °C and 5% CO2
5	Glycolate addition	1 μl/well	2 mM glycolate in media, pH 7.4
6	Incubation	2 days	at 37 °C and 5% CO2
7	ATP reagent addition	3 μl/well	freshly made
8	Incubation	10 min	at 37 °C and 5% CO2
9	Plate reading	luminescence mode	ViewLux microplate reader

**Table 2:** Confirmed HTS hit compounds.

Cell line	Compound ID	Compound name	IC50 (μM)	Max inhibition (%)	IC50* (nM)	Library
GO	NCGC00016336-01	Syrosingopine	2.3	28.7	N/A	NPC
AGT-152	NCGC00016336-01	Syrosingopine	2.5	51.5	N/A	NPC
GO	NCGC00090755-02	Potassium dichromate	7.2	92.5	86.9	NPC
AGT-152	NCGC00090755-02	Potassium dichromate	6.3	113.9	N/A	NPC
GO	NCGC00091922-02	Sodium dichromate	11.7	93.8	128.4	NPC
AGT-152	NCGC00091922-02	Sodium dichromate	9.5	132.1	N/A	NPC
GO	NCGC00094630-01	Colistimethate sodium	8.3	76.3	N/A	NPC
AGT-152	NCGC00094630-01	Colistimethate sodium	9.7	104.3	N/A	NPC
GO	NCGC00015956-05	SB 222200	20.8	37.1	N/A	LOPAC
AGT-152	NCGC00015956-05	SB 222200	29.4	94.1	N/A	LOPAC
GO	NCGC00095150-01	Telmisartan	27.4	19.9	N/A	NPC
AGT-152	NCGC00095150-01	Telmisartan	47.5	73.5	N/A	NPC

Cell line GO: CHO-GO; cell line AGT-152: CHO-GO/AGT-152. *compounds dissolved in distilled water. Signal of untransformed CHO WT cells added with DMSO was defined as 100% response. Signal of CHO-GO or CHO-GO/AGT-152 cells added with DMSO or water were defined as their respective 0% response. NCGC00015956 was obtained from Sigma-Aldrich (#S-5192, NK3 inhibitor).
